# Supplementary material for: Entropic repulsion of cholesterol-containing layers counteracts bioadhesion
Source: Nature. 2023 Jun 21;618(7966):733–9. doi: 10.1038/s41586-023-06033-4 (PMC10284698; doi:10.1038/s41586-023-06033-4)
Supplement: Supplementary file 1 — This file contains Supplementary Figs. 1–8, Tables 1–3, Notes 1–4 and References. [file 41586_2023_6033_MOESM1_ESM.pdf]

nature portfolio

https://doi.org/10.1038/s41586-023-06033-4

Supplementary information

Entropic repulsion of cholesterol-containing layers counteracts bioadhesion

In the format provided by the authors and unedited

Entropic repulsion of cholesterol-containing layers counteracts bioadhesion

Jens Friedrichs, Ralf Helbig, Julia Hilsenbeck, Prithvi Raj Pandey, Jens-Uwe Sommer, Lars David Renner, Tilo Pompe, and Carsten Werner*

Leibniz Institute of Polymer Research Dresden, Max-Bergmann Center for Biomaterials; Leipzig University; TU Dresden, Excellence Cluster Physics of Life

*Corresponding author. Email: werner@ipfdd.de

Supplementary Figures

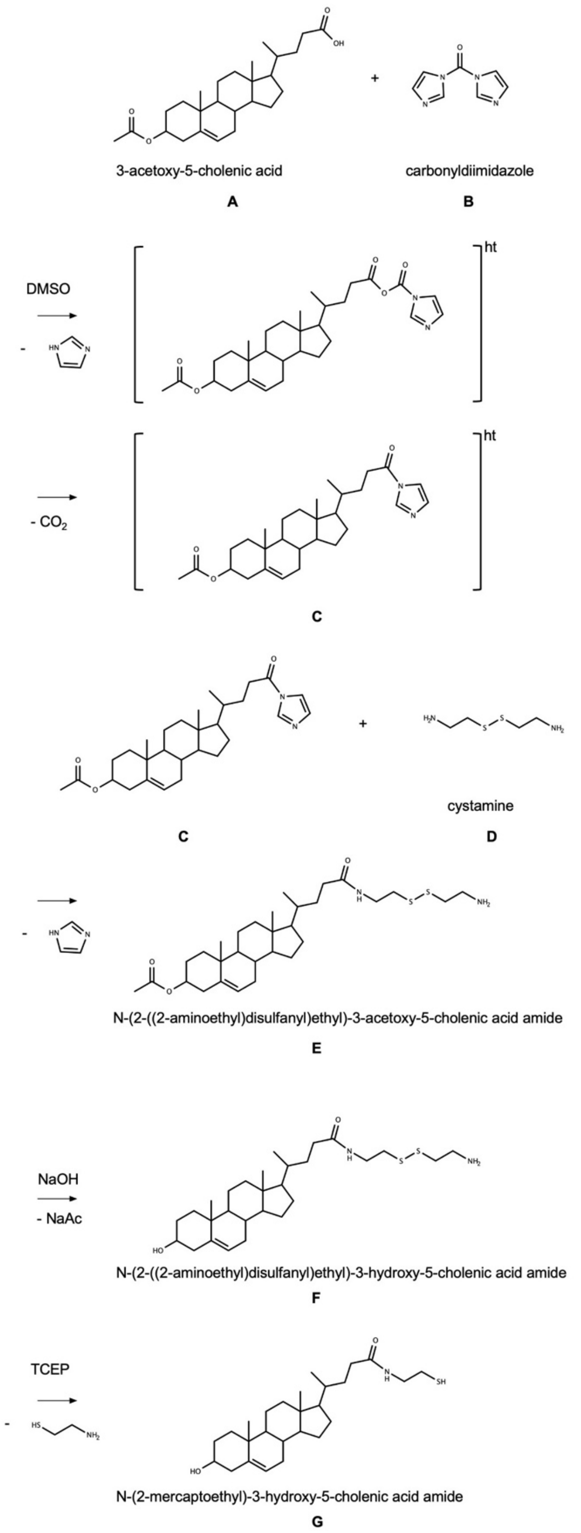

Supplementary Fig. 1 Reaction scheme for the thiolation of cholenic acid. For details, see Methods.

a

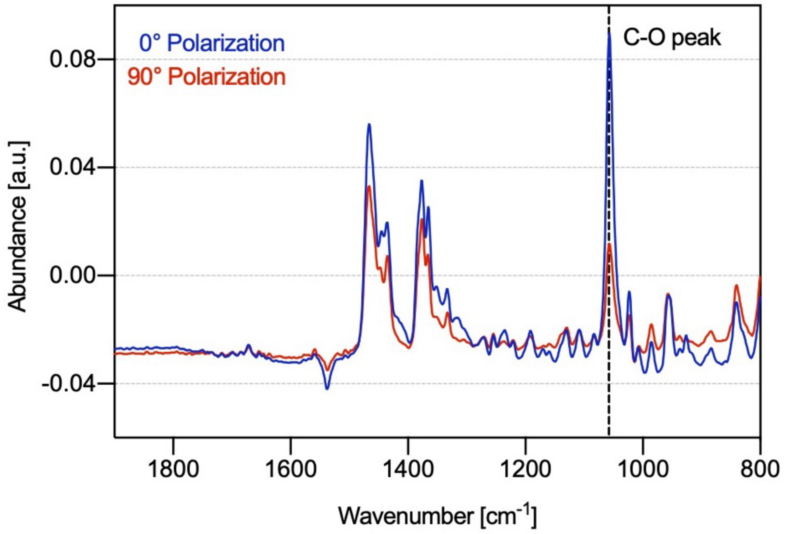

b

$$R = \frac{E_x^2}{E_y^2} + 2\frac{E_z^2\overline{\langle cos^2\Theta\rangle}}{E_y^2\left(1-\overline{\langle cos^2\Theta\rangle}\right)}$$

c

	Ex	Ey	Ez	R	*θ*	α
Cholesterol SCL	1.409	1.476	0.684	3.33	23°	23°

Supplementary Fig. 2 ATR-FTIR analysis of cholesterol SCLs. (a) Data represent p- (0°) and s- (90°) polarised ATR-FTIR spectra. Dichroic effects were observed with significant differences of the p- and s-polarized bands at the C-O (1059 cm-1) peak position. Dichroic ratios ($R = A_p/A_s$, with A representing the band intensity, p indicating p-polarization, and s indicating s-polarization) of R > 5, which are significantly different from the isotropic ratio (R = 1 - 1.5), suggest a high out-of-plane orientation with respect to the surface in the region of the cholesterol headgroups (OH-groups[1,2]). This indicates highly ordered cholesterol molecules arranged perpendicular to the substrate plane (as shown in [1]). The molecular angles of the transition dipole moments were calculated based on the measured dichroic ratios using the formula in ***(b)***.[3] There is a direct relationship between the dichroic ratio R and the Θ angle between the transition dipole moment of a given IR band and the z-axis (i.e. surface normal) of the given substrate. Ex, Ey, Ez are the relative electric field components of the evanescent IR wave, which can be calculated based on the Fresnel equations. Applying the formula in (b) to dichroic ratios measured for cholesterol SCLs, an Θ angle was obtained ***(c)***. Assuming an angle of 0° of the C-O bond with respect to the cholesterol molecular axis, an estimation of the tilt angle of cholesterol molecules with respect to the z-axis can be obtained, which in this case is identical to the Θ value. A relatively small Θ angle was obtained, which suggests a high z-axial orientation of the cholesterol molecules with respect to their molecular axis. Based on the layer thickness data (Supplementary Fig. 3a), the hypothesis can be made that cholesterol-SCLs are composed of numerous molecular layers. Considering the hydrophobic advancing contact angles of cholesterol SCLs (Extended Data Fig. 4a), it can be assumed that the hydrocarbon tail of the outer cholesterol layer is initially oriented to the air/liquid interface, while the OH-groups are oriented to the interior. A multi-lamellar layer formation composed of stacked bilayers, where hydrophilic OH-groups point to each other, is likely. The formation of these highly ordered and oriented cholesterol molecule layers is assumed to occur by self-assembly processes during spin-coating.

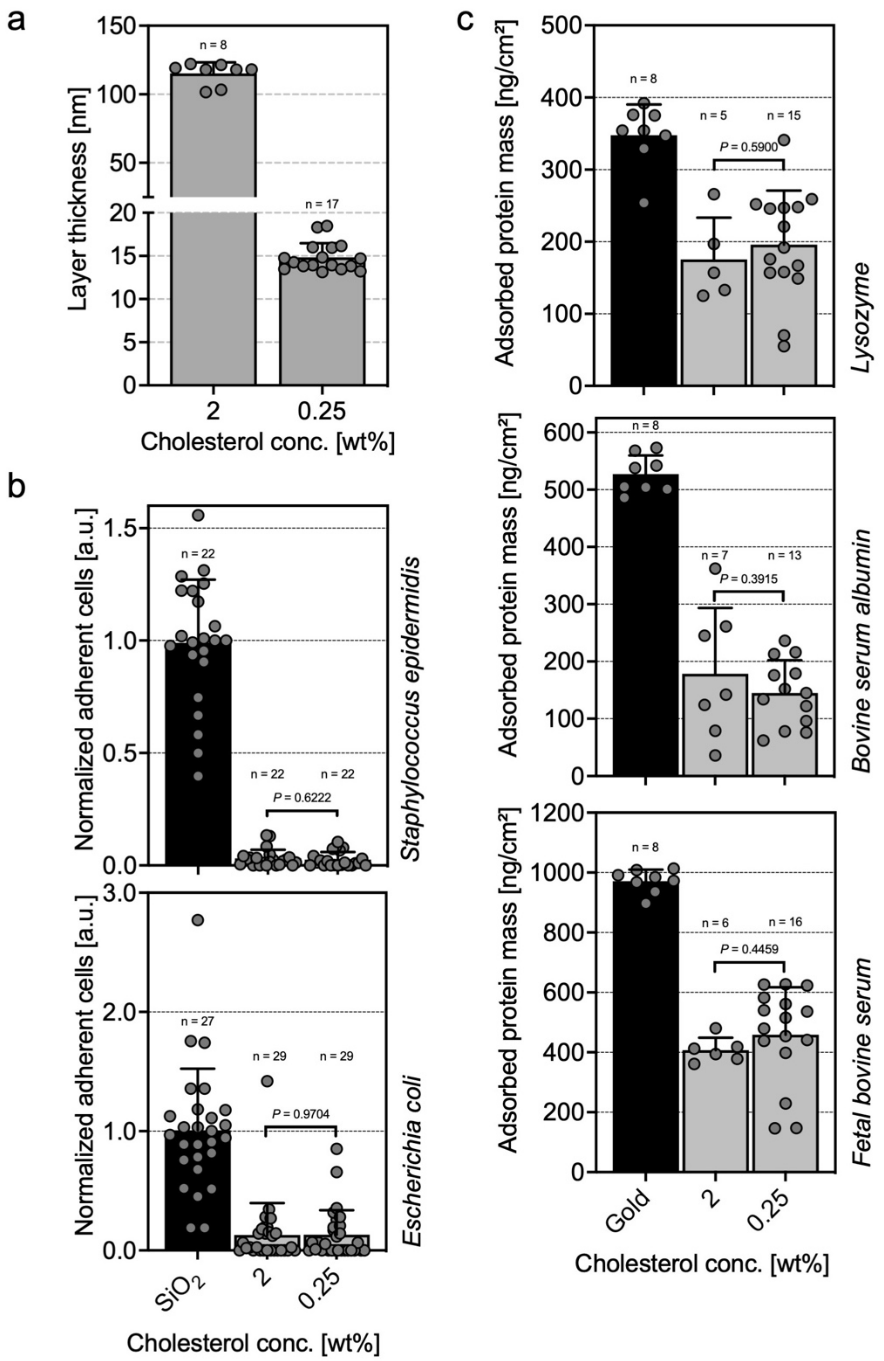

Supplementary Fig. 3 Bioadhesion characteristics of cholesterol SCLs of different layer thickness. Cholesterol SCLs were prepared from solutions containing 0.25 wt% and 2 wt% cholesterol by spin-coating. **(a)** The thickness of the resulting layers was determined by ellipsometry. **(b)** Normalised adherent cell density of *S. epidermidis* and *E. coli*. Data are normalised to the average adherent cell density on the control SiO_2 substrate. **(c)** Adsorbed amount of lysozyme, bovine serum albumin and fetal bovine serum as determined by quartz crystal microbalance measurements. No influence of the cholesterol layer thickness on the number of adherent bacterial cells or the adsorbed amount of protein was found. In all graphs, the mean + standard deviation are shown. The number of observations (n) is indicated. *P* values were determined using unpaired t-tests.

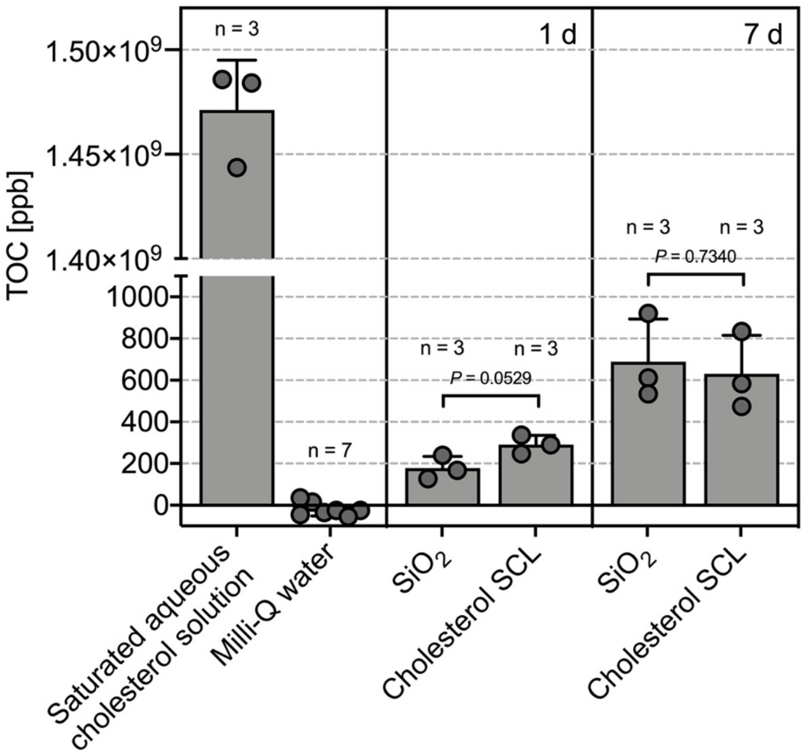

Supplementary Fig. 4 Stability of cholesterol SCLs in Milli-Q water. The total organic carbon (TOC) content of Milli-Q water was measured after incubation of cholesterol SCLs for one (1 d) and seven days (7 d). Pure Milli-Q water and Milli-Q water incubated with cleaned SiO_2 substrates served as negative controls. A saturated aqueous cholesterol solution (positive control) was prepared by dissolving 2 mg/ml cholesterol in Methanol. The dissolved cholesterol was precipitated by adding Milli-Q water, the methanol was boiled out, the solution was passed through a sterile filter, and the filtered solution was used for TOC measurements. For both time points, the TOC content of the Milli-Q water incubated with cholesterol SCLs was not significantly different from the corresponding control (i.e. cleaned SiO_2 substrate incubated with Milli-Q water). The time-dependent systematic increase of the measured values can be attributed to unavoidable contamination during the storage of the samples. The mean and + standard deviation are shown. The number of observations (n) is indicated. *P* values were determined using unpaired t-tests.

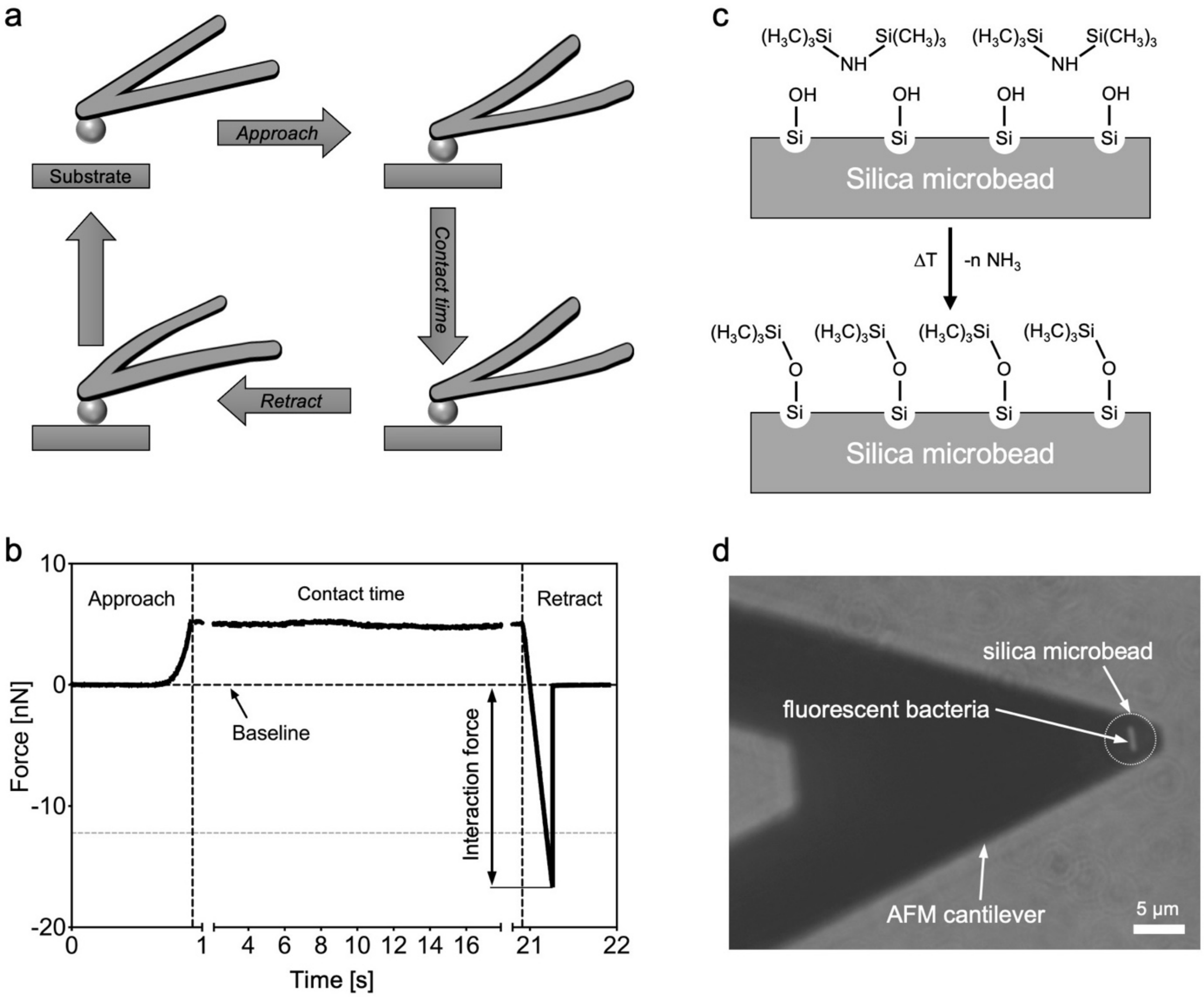

***Supplementary Fig. 5 Principle of* atomic force microscopy-based force spectroscopy measurements. (a)** Schematic illustration of a colloid probe force spectroscopy measurement. The AFM cantilever (modified with a ∅ 10µm SiO_2 colloid probe) is moved toward the sample surface, pressed onto the surface with a defined contact force, and held in this position for a variable contact time. The cantilever is then retracted until completely separated from the sample surface. During retraction of the cantilever, the interaction forces between the AFM cantilever and sample surface are detected (see (b)). AFM-based single-cell force spectroscopy experiments use the same principle. The difference is that a single bacterial cell is immobilised at the lower apex of the colloid probe before measurements (see (d)). **(b)** Exemplary force-time graph recorded during an AFM-based colloidal probe measurement of cholesterol SCLs. The AFM was operated in constant height mode during measurements. During contact (between the colloidal probe and the cholesterol surface), the deflection of the AFM cantilever was monitored and remained constant (i.e. the colloidal probe did not indent into the cholesterol SCL). Identical results were obtained in single-cell force spectroscopy experiments. **(c)** Schematic representation of the functionalisation scheme of the colloid probe with the hydrophobic silane hexamethyldisilazane. **(d)** The phase contrast image with a fluorescence image overlay shows a single *E. coli* cell immobilised on a silica microbead (attached to an AFM cantilever). This setup was used to perform single-cell force spectroscopy measurements. Further information on the experimental setup can be found in the Methods and a previous publication.[4]

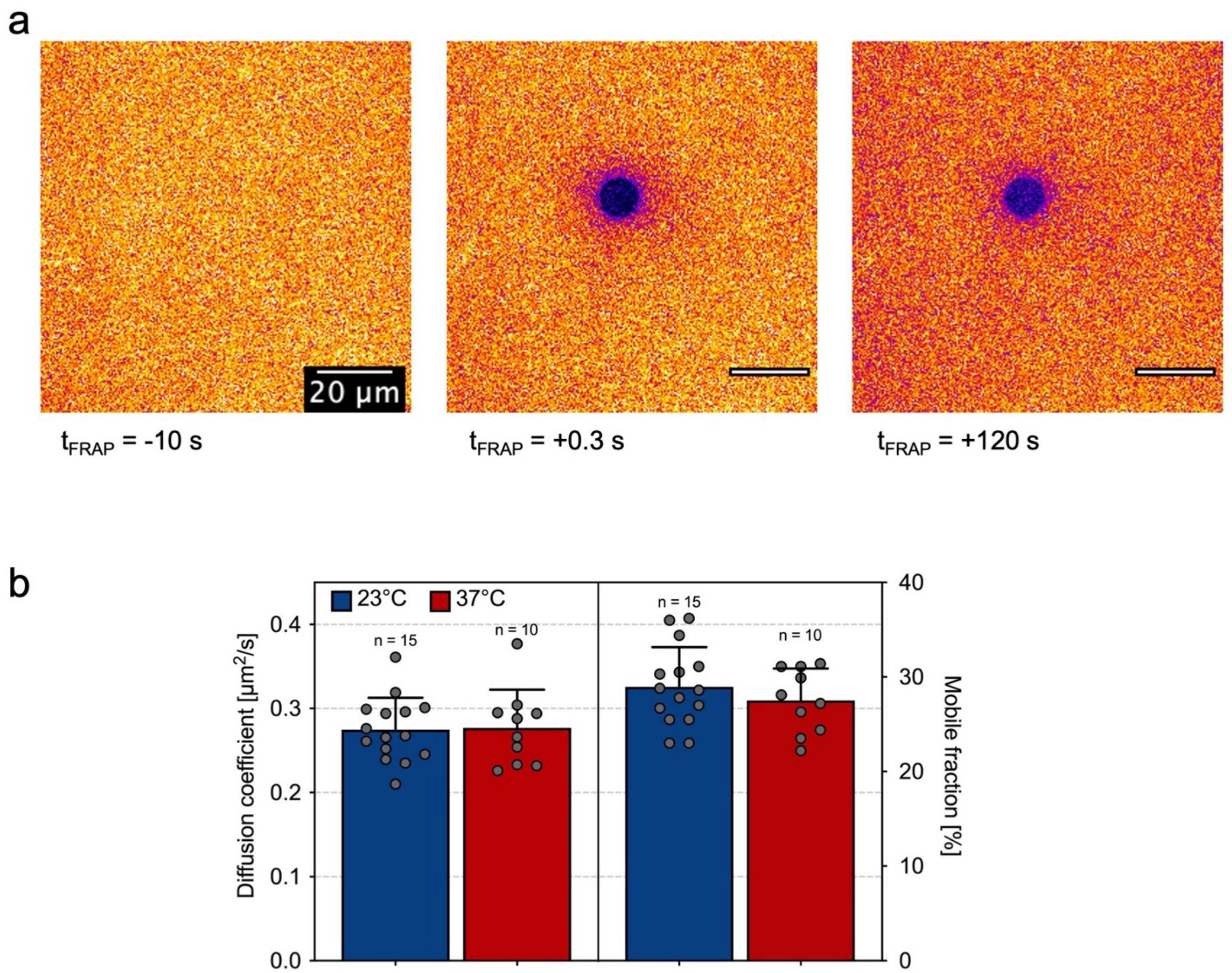

Supplementary Fig. 6 Fluorescence recovery after photobleaching (FRAP) analysis of cholesterol SCLs. **(a)** Example of a FRAP experiment on cholesterol SCLs (+1 wt% NBD-cholesterol) before bleaching, immediately after bleaching (+0.3 s), and 120 s after bleaching. **(b)** Diffusion coefficients and mobile fractions of NBD-cholesterol at 23°C and 37°C, indicating mobility in cholesterol SCLs. The number of observations (n) is indicated.

Supplementary Note 1

Analysis of the time and temperature dependence of interaction forces between the lipid layer and colloid particles or single bacterial cells

Different kinetic models were applied to analyse the AFM-based force spectroscopy data to quantify the interactions between cholesterol SCLs and colloidal probes or individual bacterial cells. The interaction force was assumed to be directly correlated to the surface concentration of the formed bonds σ. While first-order and second-order kinetics do not match the observed time dependence, third-order kinetics ($d\sigma/dt = -k \cdot \sigma^3$) fit the data with a similar time constant, k, for both colloid particle and bacterial cell adhesion.

Supplementary Note 2

Analysis of the temperature dependence of protein adsorption to lipid layers

The measured maximum mass of adsorbed lysozyme on a surface was estimated (asymptotic fit to the concentration-dependent lysozyme adsorption on gold at 20 °C; **Supplementary Fig. 7**) to be 443 ng/cm². All other measured amounts (**Fig. 3a**) were taken as fractions (q) of this maximum amount, with $0 < q < 1$ (0-100%), acquired by the method shown in **Supplementary Fig. 7**. At adsorption equilibrium,[5] the ratio of the adsorption (k_A) and desorption (k_D) constants is related to the free energy of adsorption (ΔG) by:

$$K = \frac{k_A}{k_D} = e^{\frac{-\Delta G}{RT}} = \frac{q}{c^{*}(1-q)} \qquad (1)$$

With the concentration c^* ($c^* = 6.8\cdot10^{-6}$, derived from the lysozyme concentration of 100 µg/mL, a molar mass of 14.7 kDa, and normalisation to a standard concentration of 1 M), we calculated the corresponding values of ΔG (**Supplementary Note 1** and **Extended Data Fig. 5c**). With this derivation of ΔG, we calculated the different slopes of ΔG(T) (providing ΔS of the protein adsorption) by a linear fit according to Equation (2):

$$\Delta G(T) = \Delta H - T\cdot\Delta S \qquad (2)$$

Within this first-principle approach, the enthalpy (ΔH) and the entropy (ΔS) of protein adsorption were assumed to be invariant across the investigated temperature range (15 – 40 °C). An entropic repulsion barrier of –200 ± 60 $J\cdot mol^{-1}\cdot K^{-1}$ for the adsorption of lysozyme to cholesterol SCLs was determined from the difference between ΔS of the adsorption to cholesterol SAMs and SCLs. This analysis provides the entropic repulsion of proteins at cholesterol SCLs despite additional entropic contributions associated with conformational changes and interactions with water molecules. The statistical uncertainty (standard error) of the linear fits of ΔG(T) (see **Supplementary Table 1**) was used to calculate the standard error of the determined entropic repulsion (sum of absolute values of the standard error of ΔS_{chol} and $\Delta S_{Au/SAM}$). A statistical F-test (OriginPro 2020) indicated the significantly different slope of ΔG(T) for the adsorption of lysozyme to cholesterol SCLs (**Supplentary Fig. 8**). The quantification of the entropic repulsion from adsorption data of larger, globular proteins is associated with higher statistical uncertainty. This results from a greater scatter in the adsorbed amounts due to their different interaction modes and /or adsorption-induced conformational changes. However, the fact that first-principle thermodynamics provides a dominant contribution of entropic repulsion to the overall free energy balance demonstrates the importance of the discovered effect.

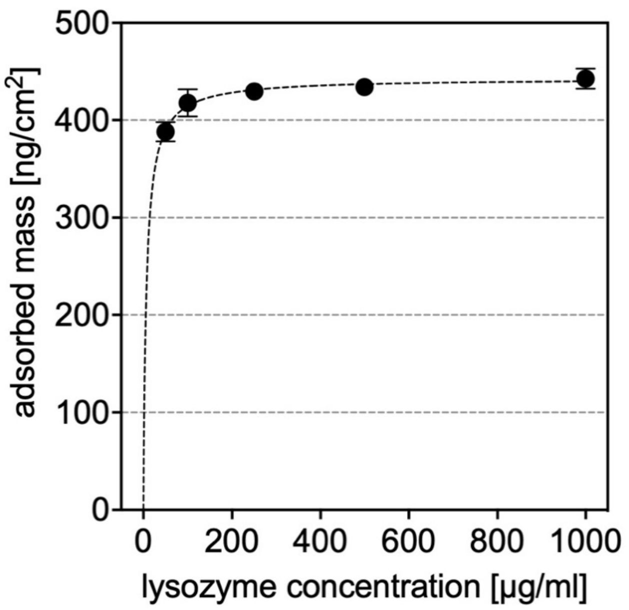

Supplementary Fig. 7 **Estimate of the partial adsorption of lysozyme at c = 100 µg/ml.** The adsorption of lysozyme at concentrations of 50, 100, 200, 500 and 1000 µg/mL on gold substrates was quantified by quartz crystal microbalance (QCM) measurements (n = 8). A hyperbolic asymptotic fit (f(x)=a(1–1/(1+c*x)^(1/d), R^2=0.99) was used to calculate the full coverage, resulting in a partial coverage of 94.3% at 100 µg/mL. All other datasets of the QCM measurements of lysozyme adsorption were correlated to this value to estimate their respective fractions of full coverage (q) in the derivation of the temperature dependent values of ΔG using Equation (1).

		Cholesterol SCL		Thiocholesterol SAM		Thiocholenic acid SAM		Au reference	
T [K]	ΔG	[kJ/mol]	SE	[kJ/mol]	SE	[kJ/(mol)]	SE	[kJ/mol]	SE
288		–30.6	1.9	–31.0	0.8	–31.5	1.3	–34.5	0.8
293		–30.0	1.5	–31.4	1.5	–32.8	1.3	–35.7	1.1
298		–30.8	0.7	–31.3	0.3	–34.3	2.1	–36.6	0.7
303		–30.6	1.8	–32.0	1.3	–33.4	0.5	–35.5	0.4
308		–29.5	0.7	–33.1	1.1	–33.9	3.6	–36.8	1.4
313		–28.7	1.1	–34.3	1.4	–35.0	2.1	–38.3	0.8
	Slope (=ΔS) [J/(mol*K)]	85	29	–112	35	–112	27	–113	49

Supplementary Table 1 **ΔG and ΔS for lysozyme adsorption.** The ΔG data were obtained using Equation (1) and the calculated maximum protein coverage fitted from data shown in **Supplementary Fig. 7**. ΔS was derived with a linear fit of ΔG(T) according to Equation 2, see **Extended Data Fig. 5c**. Statistical uncertainty is given as standard error (SE).

The temperature dependent adsorption of bovine serum albumin and fibrinogen to cholesterol SCLs, thiocholesterol SAMs, thiocholenic acid SAMs and gold was quantified based on QCM experiments **(Extended Data Fig. 5a,b)**, too. Equivalent to the method used for lysozyme, the approximation of a maximal surface coverage provides ΔG values according to Equation (1) (Supplementary Table 2 & 3). The linear fit of ΔG(T) according to Equation (2) allowed again the first-principle derivation of the entropic contribution to the free energy balance of the adsorption ΔS (Supplementary Table 2 & 3). Supplementary Fig. 8 also displays these slopes of ΔS for cholesterol SCL and the controls thiocholesterol or thiocholenic SAMs and gold with a statistical F-Test indicating the significantly different slope of cholesterol SCL in comparison to the controls for bovine serum

albumin and fibrinogen. The entropic barriers of cholesterol SCL against the adsorption of bovine serum albumin and fibrinogen (again calculated from the difference of DS of cholesterol SCL to the controls) were derived to -110 ± 60 and -70 ± 30 J·mol–1·K–1, respectively. The statistical uncertainty (standard error) was again calculated by the sum of absolute values of the standard error of ΔSchol and ΔSAu/SAM.

		**Cholesterol SCL**		**Thiocholesterol SAM**		**Thiocholenic acid SAM**		**Au reference**	
T [K]	*ΔG*	[kJ/mol]	SE	[kJ/mol]	SE	[kJ/(mol)]	SE	[kJ/mol]	SE
288		−33.4	1.4	−36.0	1.7	−35.8	1.3	−42.1	10.6
293		−32.2	1.5	−35.9	1.7	−36.5	2.2	−37.9	1.2
298		−32.4	0.8	−37.0	0.9	−38.1	1.3	−38.0	1.2
303		−32.4	1.7	−36.9	1.7	−36.1	1.2	−45.3	7.8
308		−32.4	0.9	−38.6	1.7	−37.3	0.4	−43.8	2.1
313		−31.0	1.6	−37.5	1.1	−37.5	0.6	−42.7	5.3
	Slope (=*ΔS*) [J/(mol*K)]	48	26	−69	28	−51	33	−345	103

***Supplementary Table 2 ΔG* and *ΔS* for bovine serum albumin adsorption.**

		**Cholesterol SCL**		**Thiocholesterol SAM**		**Thiocholenic acid SAM**		**Au reference**	
T [K]	*ΔG*	[kJ/mol]	SE	[kJ/mol]	SE	[kJ/(mol)]	SE	[kJ/mol]	SE
288		−37.5	0.5	−40.4	0.8	−39.0	0.1	−42.0	2.5
293		−37.4	1.3	−39.9	1.1	−40.2	0.5	−43.4	3.9
298		−38.1	1.4	−41.4	1.2	−40.2	0.3	−43.5	4.5
303		−38.0	1.3	−42.4	2.1	−41.2	0.3	−43.3	3.4
308		−38.1	0.4	−43,04	1.2	−41.4	0.1	−44.7	3.4
313		−38.8	0.3	−42,90	1.1	−41.6	0.3	−46.0	2.8
	Slope (=*ΔS*) [J/(mol*K)]	−49	10	−120	23	−119	6	−144	30

***Supplementary Table 3 ΔG* and *ΔS* for fibrinogen adsorption.**

Withthisfullerrorpropagation, asignificant andquantifiablerepulsiveentropic contribution was obtained for the adsorption of the large and conformationally 'soft' proteins bovine serum albumin and fibrinogen (see Supplementary Fig. 8). As the slope of ΔG is positive for lysozyme and bovine serum albumin, the entropic repulsion dominates the energy balance. In fibrinogen adsorption, this entropic repulsion is still effective but no longer dominant. Accordingly, the adsorption of this by far largest and softest protein is associated with a higher gain of enthalpy by deformation and/or a gain of entropy due to conformational changes. Additionally, it is important to note, although systematic errors in the QCM measurements and biased values of the maximum protein coverage could be sources for altered absolute values in **Supplementary Fig. 8**, this would not change the derived differences between cholesterol SCLs and the control substrates, as relative values were used to derive the entropic repulsion barrier ΔS of cholesterol SCL.

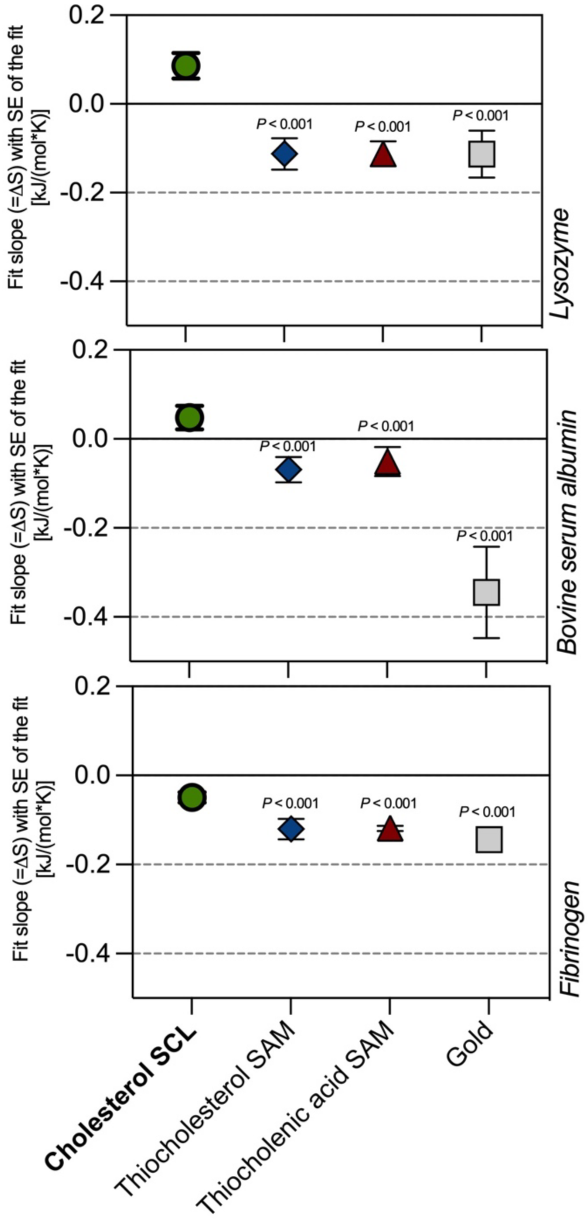

Supplementary Fig. 8 **Statistical comparison of the fitted slopes of ΔG, i.e. ΔS.** Fitted slopes of ΔG(T) (= ΔS) are shown with error bars indicating standard errors of the fit. P values were determined using statistical F-tests (OriginPro 2020). All conditions were compared to cholesterol SCLs.

Supplementary Note 3

Geometric estimation of entropic effects upon lysozyme adsorption to cholesterol SCLs

The crystal structure of cholesterol layers is described as triclinic, with angles close to 90°, a flattened shape (a=14.2 Å, b=34.2 Å, c=10.5 Å, α=94.6°, β=90.7°, and γ=96.3°), and each unit cell containing eight cholesterol molecules in a smectic-like arrangement.[7] From this structure, the surface density of cholesterol in multilayers can be derived depending on the molecular orientation considered, i.e. the surface density is approximately 2.68 nm^{-2} for vertical orientation and approximately 0.82 nm^{-2} for horizontal orientation. When in contact with water, cholesterol is oriented mostly perpendicular to the interface (**Fig. 3b**). From these dimensional considerations, one can derive that a single cholesterol molecule in a parallel orientation to the interface roughly fits the interfacial area of three cholesterol molecules having perpendicular orientations. Hence, a switch in the orientation of cholesterol must affect at least two neighbouring molecules based on geometrical considerations. This fact is consistent with the observed third-order adsorption kinetics and led us to assume a scenario of triple-cooperative orientational fluctuation of cholesterol molecules.

The freedom of mobility of fluctuating cholesterol in the interfacial layer in this scenario corresponds to an area of the largest dimension of cholesterol (assumed to be a cuboid) equating to $A_{fluct} = a \times b/4$ = 1.19 nm². If cholesterol mobility is constrained by interaction with adsorbing albumin, its mobility is strongly decreased and the available area for fluctuations corresponds roughly to the smallest dimension of cholesterol, the cross-sectional area of the cholesterol directed to the interface: $A_{constr} = a \times c/4$ = 0.35 nm².

If we take these values as upper and lower limits for configurational states of cholesterol, respectively, the entropic penalty of the mobility restriction of cholesterol upon albumin adsorption can be estimated with $\Delta S = R \times ln(A_{constr}/A_{fluct})$ to be –10.2 $J \cdot mol^{-1} \cdot K^{-1}$. Because of the triple-cooperative mobility of the system, the orientation fluctuation of three mobile molecules is coupled, and this value should be divided by three, i.e. leading to a ΔS value of –3.3 $J \cdot mol^{-1} \cdot K^{-1}$.

Considering the shape and packing of lysozyme in an adsorbed protein layer, approximately 50 interfacial cholesterol molecules can be assumed to interact with one lysozyme, which results in an entropy penalty (ΔS) of approximately –160 $J \cdot mol^{-1} \cdot K^{-1}$ for the adsorption of lysozyme to cholesterol SCLs.[8–10]

Notably, out-of-plane fluctuations, which are commonly denoted as capillary waves, might occur as well and would result in an inverse third power behaviour of the resulting repulsive force depending on the distance to the interface.[11]

Supplementary Note 4

Details on Molecular Dynamics Simulations

System equilibration

For energy minimization, the steepest descent algorithm with position restraint on the oxygen atom on all the lipid molecules with a force constant of 1000 kJ mol^{-1} nm^{-2} was used. Then, short equilibration runs were conducted: [1] for 125 ps with position restraint on all atoms of the multilayer to allow the water and ions to relax (1 fs integration timestep), [2] for 125 ps with position restraint on O-atoms on heads of all cholesterol/stigmasterol molecules to allow the

bonds, angles and dihedrals on cholesterol/stigmasterol to relax (1 fs integration timestep), [3] for 125 ps with position restraint on oxygen atoms on heads present in the lowermost layer allowing them to move only in xy-plane (1 fs integration timestep), [4] two steps for 500 ps with position restraint on oxygen atoms on heads present in the lowermost layer allowing them to move only in xy-plane (2 fs integration timestep),[12] 20 ns run with position restraint on oxygen atoms on heads present in the lowermost layer allowing them to move only in xy-plane (2 fs integration timestep). During the first two steps of the short equilibration runs, position restraints were with a force constant of 1000 $kJ \cdot mol^{-1} \cdot nm^{-2}$. During the remaining steps for the short equilibration runs, the oxygen atoms on the cholesterol/stigmasterol molecules in the lowest layer were forced by positional constraints along the z-axis to move only in the xy-plane with a force constant of 1000 $kJ \cdot mol^{-1} \cdot nm^{-2}$ to model a fixed substrate for SCLs (**Extended Data Fig. 6a**). All bonds with hydrogen were constrained with the LINCS[13] algorithm during equilibration steps. A force-based switching method was used to smoothly switch off *van der Waals* interactions between 1.0 to 1.2 nm.[14] The walls were modelled as a direct 12-6 LJ potential with hydrophobic CTL3-atom type in charmm36 force field. The energy minimization step was conducted without the walls, and included the walls at z = 0 and z = 50 nm during all short equilibration runs. A cutoff for coulomb interactions of 1.2 nm was applied and long-range electrostatics interactions were determined with particle-mesh Ewald (PME) method adapted for slab-geometry[15] implemented in Gromacs as ewald-geometry 3dc, and applied periodic boundary conditions (PBC) along x and y directions in all the short equilibration runs. During all short equilibration runs, the refcoord-scaling option in Gromacs as "all" was used. All short equilibration runs were conducted in NVT ensemble at 303.15 K temperature with v-rescale thermostat[16] with 1 ps time constant for temperature coupling.

Production simulations

Production simulations were started from the structures obtained after equilibration. For cholesterol- and stigmasterol-containing multilayer systems without reverted molecules at the interface layer (**Extended Data Fig. 6e)**, three trajectories of 1 µs each were generated, resulting in a total of 6 trajectories. Likewise, for cholesterol- and stigmasterol-containing multilayer systems with 10%, 30%, and 50% of reverted molecules at the interface layer (**Extended Data Fig. 6e)** three trajectories of 1 µs each were generated, resulting in a total of 18 trajectories. A total of 24 trajectories with a length of 1 µs each were generated.

During production simulations, all bonds with hydrogen were constrained using the LINCS algorithm[13] and integration time-steps of 2 fs were used. Position restraints along the z-axis with a force constant of 1000 $kJ\ mol^{-1}nm^{-2}$ were applied to the oxygen atoms of lipid molecules in the lowermost layer to restrain them to move only in the xy-plane to model a solid substrate on which SCLs were constructed. As in the short equilibration runs, the force-based switching method was used to smoothly switch off van der Waals interactions between 1.0 to 1.2 nm, 1.2 nm cutoff for coulomb interactions in the production simulations. Long-range electrostatic interactions were obtained with the PME method adapted for slab-geometry implemented in Gromacs as ewald-geometry 3dc. Box dimensions during the production simulations were 7.1554 nm × 7.1554 nm × 50 nm which included vacuum above and below lipid-water systems along the z-axis. Walls at z = 0 and z = 50 nm as direct 12-6 LJ potential with hydrophobic CTL3-atom type in CHARMM36 force field, and PBC was applied along x and y directions in all the production

simulations. The refcoord-scaling option in Gromacs as com was used during production simulations. All production simulations were conducted in the NVT ensemble at 303.15 K temperature and V-rescale thermostat with a 1 ps time constant for temperature coupling.

Analysis

The gmx trjconv Gromacs tool was used to remove the pbc effect from simulation trajectories. To conduct the analyses of the pbc-treated simulation trajectories, the MDTraj tool[17] was used to read the simulation trajectories.

1. Calculation of correlation

The correlation function (C_r) represents the correlation of the orientation vector on a reference molecule (0) with the orientation vector on any molecule falling within a distance range (r) from the reference molecule.

$$C_r = \frac{\int_0^T dt\,\left[\overrightarrow{S_r}(t) - \langle\overrightarrow{S_r}\rangle\right].\left[\overrightarrow{S_0}(t) - \langle\overrightarrow{S_0}\rangle\right]}{\int_0^T dt\,\Delta\overrightarrow{S_r}^2}, \Delta\overrightarrow{S_r}^2 = \left(\overrightarrow{S_r}(t) - \langle\overrightarrow{S_r}\rangle\right)^2$$

S represents the orientation vector on a molecule and was calculated as the unit vector connecting the C17 and C3 atoms in cholesterol/stigmasterol molecules (**Extended Data Fig. 6c**). C_r was only calculated for cholesterol/stigmasterol molecules in the interfacial layer for distance ranges 0–1 nm, 1–2 nm, 2–3 nm, 3–4 nm, and 4–5 nm in each frame of MD trajectories. C_r for each S_r was calculated by integrating time patches of 0.02 µs. The mean C_r of all S_r for all the time patches in the range 0 to 1 µs were plotted in dependence on distance range r (**Fig. 3e** and **Extended Data Fig. 6e**) with error bars representing standard deviation over time patches.

2. Root mean square deviation (RMSD) of interface layer hydrophilic head in the multilayer systems without any reverted molecules in the interface layer

The RMSD of the z-component of the position of the hydrophilic head (oxygen atom) on cholesterol or stigmasterol molecules in the interfacial layer was calculated to explore the fluctuation of the interface layer in contact with water. First, the mean centre of mass of all the oxygen atoms on cholesterol or stigmasterol molecules in the interface layer in the time range of 0.5 to 1 µs was calculated in each simulation trajectory for systems without any rotated molecules at the interface layer. Then, the RMSD of the z-component of the position of each oxygen atom in the interface layer from the z-component of the mean centre of mass in each frame of the simulation trajectory was calculated in the time range 0.5 to 1 µs. From this, the mean RMSD of the z-component of the position of oxygen atoms in each frame of the three simulation trajectories was obtained in the time range of 0.5 to 1 µs. Finally, the mean RMSD over three simulation trajectories in each frame was obtained in the time range of 0.5 to 1 µs. **Extended Data Fig. 6f** shows a histogram of the RMSD created from the mean RMSD values of three simulation trajectories in all frames in the time range from 0.5 to 1 µs, as well as, a plot of the mean RMSD over 3 trajectories as a function of time for frames every 10 ns, with error bars representing standard error of mean at each point over 3 simulation trajectories.

Supplementary Video 1 Video sequence of a dynamic water contact angle measurement on the surface of a cholesterol SCL. The high advancing contact angle reflects a hydrophobic surface. Upon immediate withdrawal of the droplet (no resting period) a slightly reduced but still high receding contact angle is observed.

Supplementary Video 2 Video sequence of a dynamic water contact angle measurement on the surface of a cholesterol SCL. The high advancing contact angle reflects a hydrophobic surface. No receding of the three-phase contact line is observed when the water droplet is withdrawn after a rest period of 20 s The water droplet is then retracted. Oscillations in the three-phase contact line were observed during the 20 s rest period.

Supplementary References

1. Müller, M., Ouyang, W. & Keßler, B. Dichroic ATR-FTIR spectroscopy on oriented α-helical poly(l-lysine) multilayered with polyanions. *Spectrochim. Acta - Part A Mol. Biomol. Spectrosc.* **77**, 709–716 (2010).
2. Müller, M. Orientation of α-helical poly(L-lysine) in consecutively adsorbed polyelectrolyte multilayers on texturized silicon substrates. *Biomacromolecules* **2**, 262–269 (2001).
3. Matijašević, J., Hassler, N., Reiter, G. & Fringeli, U. P. In situ ATR FTIR monitoring of the formation of functionalized mono- and multilayers on germanium substrate: From 7-octenyltrichlorosilane to 7-carboxylsilane. *Langmuir* **24**, 2588–2596 (2008).
4. Beaussart, A. *et al.* Quantifying the forces guiding microbial cell adhesion using single-cell force spectroscopy. *Nat. Protoc.* **9**, 1049–1055 (2014).
5. Latour, R. A. Thermodynamic perspectives on the molecular mechanisms providing protein adsorption resistance that include protein-surface interactions. *J. Biomed. Mater. Res. - Part A* **78**, 843–854 (2006).
6. Horbett, T. A. Fibrinogen adsorption to biomaterials. *J. Biomed. Mater. Res. A* **106**, 2777 (2018).
7. Shieh, H. S., Hoard, L. G. & Nordman, C. E. Crystal structure of anhydrous cholesterol [39]. *Nature* vol. 267 287–289 (1977).
8. Klose, T., Welzel, P. B. & Werner, C. Protein adsorption from flowing solutions on pure and maleic acid copolymer modified glass particles. *Colloids Surfaces B Biointerfaces* **51**, 1–9 (2006).
9. Cordeiro, A. L. *et al.* Protein adsorption dynamics to polymer surfaces revisited—A multisystems approach. *Biointerphases* **14**, 051005 (2019).
10. Hamill, A. C., Wang, S. C. & Lee, C. T. Probing lysozyme conformation with light reveals a new folding intermediate. *Biochemistry* **44**, 15139–15149 (2005).
11. Lipowsky, R. Generic interactions of flexible membranes. in *Handbook of Biological Physics* (eds. Lipowsky, R. & Sackmann, E. B. T.-H. of B. P.) vol. 1 521–602 (North-Holland, 1995).
12. Jefferys, E., Sands, Z. A., Shi, J., Sansom, M. S. P. & Fowler, P. W. Alchembed: A Computational Method for Incorporating Multiple Proteins into Complex Lipid Geometries. *J. Chem. Theory Comput.* **11**, 2743–2754 (2015).
13. Hess, B., Bekker, H., Berendsen, H. J. C. & Fraaije, J. G. E. M. LINCS: A Linear Constraint Solver for molecular simulations. *J. Comput. Chem.* **18**, 1463–1472 (1997).
14. Steinbach, P. J. & Brooks, B. R. New spherical-cutoff methods for long-range forces in macromolecular simulation. *J. Comput. Chem.* **15**, 667–683 (1994).
15. Yeh, I. C. & Berkowitz, M. L. Ewald summation for systems with slab geometry. *J. Chem. Phys.* **111**, 3155–3162 (1999).
16. Bussi, G., Donadio, D. & Parrinello, M. Canonical sampling through velocity rescaling. *J. Chem. Phys.* **126**, (2007).
17. McGibbon, R. T. *et al.* MDTraj: A Modern Open Library for the Analysis of Molecular Dynamics Trajectories. *Biophys. J.* **109**, 1528–1532 (2015).
